# Outcomes of and Satisfaction with the Inflatable Penile Prosthesis in the Elderly Male

**DOI:** 10.1155/2012/240963

**Published:** 2012-05-29

**Authors:** Humberto G. Villarreal, LeRoy Jones

**Affiliations:** ^1^Department of Urology, University of Texas Health Science Center at San Antonio, 7703 Floyd Curl Drive, P.O. Box 7845, San Antonio, TX 78229-3900, USA; ^2^Urology of San Antonio, 7909 Fredericksburg Road, Suite 110, San Antonio, TX 78229, USA

## Abstract

*Objective*. To determine the outcomes of and satisfaction with the multi-component inflatable penile prosthesis (IPP) in the elderly male (age >71). *Methods*. Using a chart review and telephone survey, we retrospectively assessed patients who underwent IPP or combined IPP/artificial urinary sphincter (AUS) from 2004–2006. *Results*. We identified 56 patients that underwent IPP (48) or IPP/AUS (8). The age range was 71–86 (mean 74.3) at the time of surgery, with a follow-up range of 0.5–2.4 years (mean 1.5). The overall complication rate was 3.8% (2 of 56) with one device removed for infection and a second patient requiring exploration for a postoperative hematoma. The telephone interview was conducted with 35 of 56 patients. Patients rated ease of use (a scale from 1–5, 5 meaning very easy) and overall satisfaction (a scale of 1–5, 5 meaning very satisfied) at an average of 4.1 and 4.3, respectively. IPP usage varied from 0–7 times per month (mean 3.3). 32 of 35 patients (91%) said they would undergo the procedure again. *Conclusion*. Our review demonstrates that the IPP is well tolerated in the elderly male population, who report a high degree of satisfaction and ease of use with this device.

## 1. Introduction and Objective

Erectile dysfunction is highly prevalent in our society and this prevalence will increase with age. Life expectancy continues to increase with US men now living greater than 75 years [1]. It is estimated that up to 70% of males aged 70 have erectile dysfunction. The advent of oral phosphodiesterase type 5 inhibitors has allowed many men to regain normal sexual function and has placed the treatment of erectile dysfunction at the forefront of men's health issues. When conservative therapy fails or when patients are not candidates for oral therapy, the multicomponent inflatable penile prosthesis (IPP) remains the gold standard of treatment. The safety and efficacy of the IPP has been well documented, but in spite of this, urologists may be reluctant to offer an IPP to older patients due to various concerns, including those regarding impaired dexterity of older patients and their ability to operate an inflatable device. There is little published data specifically examining results of the IPP in elderly men. The objective of this study was to determine the outcome and satisfaction of the inflatable penile prosthesis in the elderly male (age >70 years).

## 2. Methods

We conducted a single-center, two-phase analysis of patients over age 70 that underwent either IPP or combined IPP/Artificial Urinary Sphincter (AUS) from 2004 to 2006. All patients received routine preoperative counseling regarding risks, benefits, and realistic expectations of the surgery. Preoperative medical clearance was obtained if deemed necessary by the surgeon. All penile implants were from the AMS 700 series with Inhibizone and tactile pump. Our routine is to administer intravenous cefazolin preoperatively and perform a 10-minute surgical prep with betadine. However, since conclusion of this study, we have modified our surgical prep to chlorhexidine gluconate due to its recently proven superiority to povidone-iodine for surgical site antisepsis [2]. All implants were performed by a single experienced prosthetic surgeon (LAJ). The initial phase consisted of a retrospective chart review and data collection with regards to patient age at implantation, type of surgery (IPP or dual IPP/AUS implantation), length of follow-up, and complications. The second phase involved a voluntary telephone survey conducted by a neutral party to assess ease of use and overall satisfaction (see the Appendix).

## 3. Results

**Figure 1:**
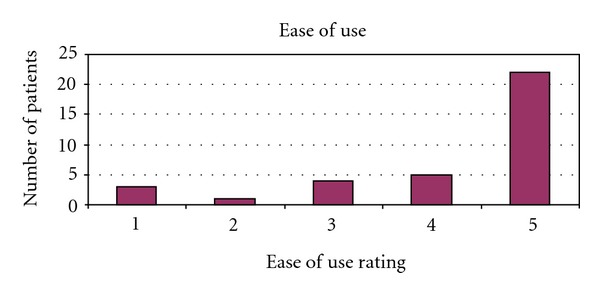


**Figure 2:**
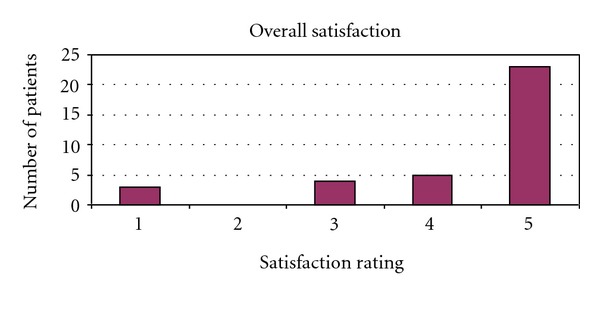


**Figure 3:**
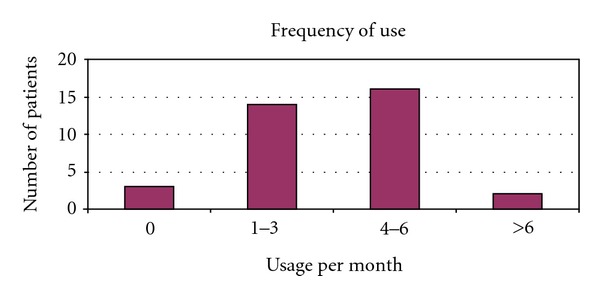


We identified 56 men that underwent either IPP (48) or IPP/AUS (8). Age ranged from 71 to 86 (mean 74.3) years of age at time of surgery. The postoperative follow-up range was 0.5–2.4 years (mean 1.5). In this group of patients, one device (IPP only patient) was removed for infection 8 months after implantation (infection rate 1.7%). Another patient had a postoperative hematoma requiring exploration, resulting in an overall complication rate of 3.4% (2 of 56). The telephone interview was performed on 35 of the 56 patients for a response rate of 62%. One patient had died and the remaining patients were unable to be contacted resulting in a nonresponse rate of 38% (21 of 56). No patient declined participation. Patients rated ease of use (on a scale from 1 to 5, with 5 meaning very easy to use) at an average of 4.1 (Figure 1) with difficulty inflating the device sited as a particular issue impairing use. Patients also rated overall satisfaction (on a scale from 1 to 5, with 5 meaning very satisfied) at an average of 4.3 (Figure 2). 28 of 35 patients (80%) rated their overall satisfaction 4 or 5. 32 of 35 patients (91%) rated their overall satisfaction 3 or higher. The most common complaint was dissatisfaction with penile length, reported by 3 of the responding patients, which is a complaint consistent with other previously published studies in the urologic literature [3]. Patients reported frequency of use of the IPP at 0–7 times per month (mean 3.3) (see Figure 3). 32 of 35 patients (91%) said that they would choose to undergo the procedure again. Those declining noted poor rigidity (2) or partner dissatisfaction (1). 29 of 35 patients (83%) stated that they would recommend this procedure to a friend.

## 4. Discussion

The safety and efficacy of the IPP has been well documented with long-term follow-up. Carson and associates from the AMS 700 CX study group evaluated patient satisfaction outcomes in 372 men [4]. They found that at a median follow-up of 47 months, 79% used their prosthesis at least twice monthly and that 88% would recommend this procedure to others. The mean age of their population was 57.6 years. Levine et al. reviewed 131 men with a mean age of 56.8 years implanted with the 2-piece Ambicor inflatable penile prosthesis [5]. They report an overall satisfaction rate 90% of patients and 82% of partners using the Erectile Dysfunction Inventory of Treatment (EDITS) questionnaire. 93% of their patients and 90% of partners would recommend the implant to others. A prospective approach using pre- and postoperative EDITS as well as IIEF scores was by Mulhall and colleagues who evaluated 96 men with a mean age of 56 years [6]. They confirmed statistically significant improvement over baseline in both inventories at 12 months after IPP placement.

There are additional studies that support the overall efficacy of the IPP, but there is little that examines this issue exclusively in an elderly population. This is an important issue considering the continual increase in life expectancy and a sense that longevity should be accompanied by quality of life. Due to increasing life expectancy, the population of Americans aged 65 years or older is anticipated to double in the next 25 years [7]. As a result, we anticipate a significant increase in the number of elderly men seeking penile prosthesis implantation. Lindau et al. recently reported that elderly couples remain sexually active even into the eighth decade of life [8]. The mean age of patients in our study was 74.3 years at the time of surgery and no patient younger than 71 years old was included. Most previous studies have examined populations with an average age in the 1950s, though Goldstein and associates examined Mentor Alpha-1 prostheses in a population with a mean age of 61 in whom 89% “had fulfilled expectations” [9]. O'Connor et al. recently evaluated a group of elderly men (above age 75) who had undergone implantation of the artificial urinary sphincter and found overall success with satisfactory results in terms of continence, complications, and longevity of the device [10].

In general, results in this elderly cohort are comparable to those of younger patients. Our infection rate and overall complication rate, 1.7% and 3.4%, were low and comparable to others reported in the general population [11]. The previously cited paper by Levine notes a 7.6% complication rate, though with longer follow-up of 43 months. The decreased dexterity of elderly patients or other comorbidities such as arthritis and neuropathy might be of concern when choosing an IPP as opposed to a malleable implant. This is certainly a reasonable concern, but the average score of 4.1 (scale of 1–5, 5 being easiest) for ease of use demonstrates that age alone should not favor a malleable device over an inflatable one. Patients should be honestly evaluated based on their current functional status. Overall satisfaction was also high, with an average score of 4.3 (5 being the most satisfied), and this satisfaction was evident in the frequency of implant use. Dissatisfaction due to subjective loss of penile length was reported by 3 of 35 surveyed patients. The previously referenced study by Deveci et al. evaluated penile length alterations after penile prosthesis surgery and did not note a negative impact on stretched penile length following surgery in their cohort [3]. In fact, despite any evidence in penile length alteration, >70% of men in their study had subjective loss of penile length. We use traditional sizing and corporeal dilation techniques at the time of IPP placement. It is our practice to counsel the patient extensively regarding the anticipated appearance and function of the penis after prosthetic implant, and as a result, we find that patients with complaints of loss of penile length are in the minority. The frequency of implant use ranged from 0 to 7 with a mean of 3.3 times per month, comparing favorably with the younger group evaluated by Carson et al. where 79% of the patients used their device for intercourse at least twice monthly. For comparison purposes, about an equal percentage (86%) of our older population used their IPP at least twice monthly. Direct comparison between this group of patients and younger groups is difficult for several reasons. We used a telephone survey which lends itself to brief encounters and therefore involves a simple but invalidated instrument instead of longer validated ones such as the IIEF or EDITS. We are also keenly aware of the biases that accompany a survey. The 38% nonresponse rate may bias the results towards increased satisfaction and more positive results. Furthermore, though differences in patient satisfaction and ease of use between patients undergoing IPP compared to those undergoing dual implant (IPP/AUS) may exist, our limited sampling of patients undergoing dual implant precluded us from adequately assessing for this potential confounder. Nevertheless, we have demonstrated that even in an elderly population, the IPP is very safe and met with a high degree of patient satisfaction. Future evaluation in a prospective manner with validated instruments will further solidify this conclusion. Prospective evaluation of the partners of these elderly men would also be a valuable addition to the body of literature and would aid in better counseling of elderly patients.

## 5. Conclusions

Implantation of the inflatable penile prosthesis should not be avoided in elderly males based solely on the age of the patient. Our paper demonstrates that the IPP is well tolerated in this patient population, who report a high degree of satisfaction and ease of use with this device.

## Appendix

IPP Questionnaire

1. Ease of use:
  How would you rate the ease of use of the IPP on a scale from 1 to 5, with 1 being very difficult to use and 5 being very easy to use.
2. Overall satisfaction:
  How would you rate your overall satisfaction with the IPP on a scale from 1 to 5, with 1 being very unsatisfied and 5 being very satisfied.
3. Frequency of use:
  How often would you say that you are using the IPP on a monthly basis?
  None, 1–3 times, 4–6 times, or greater than 6 times
4. Would you recommend the IPP to a friend?
5. Would you undergo this procedure knowing what you know now about the IPP?
